# Inter-species diversity and functional genomic analyses of closed genome assemblies of clinically isolated, megaplasmid-containing *Enterococcus raffinosus* Er676 and ATCC49464

**DOI:** 10.1099/acmi.0.000508.v3

**Published:** 2023-06-12

**Authors:** Belle M. Sharon, Neha V. Hulyalkar, Philippe E. Zimmern, Kelli L. Palmer, Nicole J. De Nisco

**Affiliations:** ^1^​ Department of Biological Sciences, University of Texas at Dallas, Richardson, Texas, USA; ^2^​ Department of Urology, University of Texas Southwestern Medical Center, Dallas, Texas, USA

**Keywords:** *Enterococcus raffinosus*, complete genome, megaplasmid, genomics, UTI

## Abstract

*
Enterococcus raffinosus* is an understudied member of its genus possessing a characteristic megaplasmid contributing to a large genome size. Although less commonly associated with human infection compared to other enterococci, this species can cause disease and persist in diverse niches such as the gut, urinary tract, blood and environment. Few complete genome assemblies have been published to date for *E. raffinosus *. In this study, we report the complete assembly of the first clinical urinary *E. raffinosus* strain, Er676, isolated from a postmenopausal woman with history of recurrent urinary tract infection. We additionally completed the assembly of clinical type strain ATCC49464. Comparative genomic analyses reveal inter-species diversity driven by large accessory genomes. The presence of a conserved megaplasmid indicates it is a ubiquitous and vital genetic feature of *E. raffinosus *. We find that the *E. raffinosus* chromosome is enriched for DNA replication and protein biosynthesis genes while the megaplasmid is enriched for transcription and carbohydrate metabolism genes. Prophage analysis suggests that diversity in the chromosome and megaplasmid sequences arises, in part, from horizontal gene transfer. Er676 demonstrated the largest genome size reported to date for *E. raffinosus* and the highest probability of human pathogenicity. Er676 also possesses multiple antimicrobial resistance genes, of which all but one are encoded on the chromosome, and has the most complete prophage sequences. Complete assembly and comparative analyses of the Er676 and ATCC49464 genomes provide important insight into the inter-species diversity of *E. raffinosus* that gives it its ability to colonize and persist in the human body. Investigating genetic factors that contribute to the pathogenicity of this species will provide valuable tools to combat diseases caused by this opportunistic pathogen.

## Data Summary

The authors confirm all supporting data, code and protocols have been provided within the article or through supplementary data files. Please see the Genome Collection section in the Methods for detailed BioSample accessions for all strains used in this study. Complete genome sequences and raw read data have been deposited as part of BioProject PRJNA880513. Additional intermediate data analysis files not already included in the Supplemental Material can be found on Figshare: https://doi.org/10.6084/m9.figshare.22097240.v1 [1].

**Impact Statement**

*
Enterococcus raffinosus* has been associated with various human infections. Despite its relevance in human health, few genome sequences have been published for this species and only two are complete. Complete genome assemblies are particularly important for understanding the contribution of mobile genetic elements such as plasmids or phage to host colonization and pathogenicity. They further provide important data for studying the function of genes important to the physiology and pathogenesis of *E. raffinosus *. Unlike other members of its genus, *E. raffinosus* has been reported to possess a megaplasmid that encodes key genes, such as raffinose metabolism genes, that differentiate this species from other enterococci. Here we report the complete, closed genome assemblies of two clinically isolated *E. raffinosus* strains: Er676 – isolated from the urine of a postmenopausal patient with history of recurrent urinary tract infection, and ATCC49464 – a type strain isolated from an unknown clinical source that previously only had draft genomes published in 2013. The availability of additional complete genomes, the first of a urinary isolation source, and of a commercially available type strain, can advance both genotypic and phenotypic studies of this species and lend further knowledge to combating *E. raffinosus* infections.

## Introduction

Enterococcal urinary tract infections (UTIs) are common and pose a serious health burden, particularly in chronically ill patients [2]. Multidrug-resistant enterococci further complicate treatment of UTIs and other nosocomial infections caused by these opportunistic pathogens [3]. A member of the genus *Enterococcus *, *Enterococcus raffinosus *, although less prevalent in human infection than *E. faecalis* and *E. faecium *, was found to be associated with nosocomial infections, bacteraemia and UTIs [4–6]. Despite previous reports of an association with UTI, urinary strains of *E. raffinosus* remain largely understudied [4]. Since its identification in 1989 and distinction from the closely related *Enterococcus avium* due to its ability to metabolize raffinose, few *E. raffinosus* case reports have been published and no larger epidemiological studies have been conducted [4, 7]. Raffinose metabolism is not common among enterococci although it has been reported in other *Enterococcus* species such as *E. mundtii* and *E. casseliflavus* [8]. However, it is primarily found in *E. raffinosus* and enables *E. raffinosus* to utilize raffinose as a sole carbon source for growth and metabolism [9, 10].

High-throughput genomic studies of genetic determinants of pathogenicity and antimicrobial resistance rely heavily on the availability of high-quality genome assemblies. Closed genome assemblies are particularly important for understanding the contribution of mobile genetic elements (MGEs), such as plasmids, to genomic diversity, environmental adaptation, antibiotic resistance and pathogenicity [11]. *E. raffinosus* has been reported to possess a megaplasmid, a genetic feature uncommon within the genus *Enterococcus *, that encodes diverse accessory genes particularly involved in carbohydrate metabolism and other vital cellular functions [12]. These observations were based on the complete genome assemblies of CX012922 and F162_2 strains that were isolated from a human faecal sample and Swiss water stream environment, respectively, and were published in 2021 [12, 13].

Megaplasmids have been previously reported in several other bacterial species dating back to their initial observation in 1981 in *Rhizobium* spp. [14]. Characterized by a size larger than 350 kb, they have been found to encode antibiotic resistance, metabolic pathways and symbiotic genes [15, 16]. Distinguishing megaplasmids from secondary chromosomes relies primarily on the presence of essential genes [14]. *E. raffinosus* has been reported to encode raffinose metabolism genes on its megaplasmid, suggesting that the megaplasmid plays an important role in the metabolic plasticity of the species [12]. Interestingly, the closest relative to *E. raffinosus* reported to possess a megaplasmid is *E. faecium *, and the presence of raffinose metabolism genes has been similarly observed in *E. faecium* megaplasmids [17]. However, *E. raffinosus* megaplasmid-encoded functions beyond raffinose metabolism remain unclear. Genomic studies to resolve these functions have been limited by the availability of only two complete *E. raffinosus* genomes.

This study expands the availability of complete, closed genome assemblies of *E. raffinosus* strains. Reported herein is Er676, clinically isolated from the urine of a postmenopausal woman with history of recurrent UTI, and ATCC49464, a type strain isolated from an unknown clinical source that previously only had draft genomes published in 2013 [18]. Importantly, Er676 represents the first complete genome assembly of a urinary *E. raffinosus* strain. We further perform comparative genomics of all available assemblies of *E. raffinosus* and find that *E. raffinosus* possesses a large accessory genome contributing to its genomic diversity and environmental adaptability. We find that the megaplasmid contributes to accessory gene content, is a ubiquitous feature of the species, and is enriched in genes involved in transcription and carbohydrate metabolism. Interestingly, we find that all antimicrobial resistance genes (ARGs) identified by our analysis are chromosomally encoded and not encoded within the megaplasmid. These findings suggest that while many pathogenicity-related factors are chromosomally encoded, the megaplasmid plays a vital role in imperative cellular functions of *E. raffinosus *, thereby supporting its growth and survival.

## Methods

### Genome collection

Er676 and ATCC49464 were both sequenced as part of this study and deposited to NCBI in BioProject PRJNA880513. All relevant accessions are further described in Table 1. Additional *E. raffinosus* genomes were retrieved from NCBI for comparison. All genomes with assembly size >3 Mbp and average nucleotide identity (ANI) >5 % were included (nine in total). Draft assemblies of ATCC49464 (*SAMN02596957*, *SAMN00809111*) were excluded as they do not provide a closed genome, but they were aligned to the complete ATCC49464 assembly to confirm the assemblies are similar. Publicly available genome assemblies used include: CX012922 (*SAMN20857173*), F162_2 (*SAMN18631319*), L3_072_123G1_dasL2_072_123G1_concoct_6 (*SAMN17801356*), DSM 5633 (*SAMN03267183*), BIOML-A1 (*SAMN11945546*), K36 (*SAMN14389560*), MGYG-HGUY-01696 (*SAMEA5851200*), NBRC 100492 (*SAMD00045732*) and cftri2200 (*SAMN02299317*). N17 (*SAMN19374046*) was excluded owing to low ANI and is probably not a member of the *E. raffinosus* species. Colony537 (*SAMN17910433*) was excluded as it does not represent a full *E. raffinosus* genome.

**Table 1.** Assembly accessions and parameters of complete *E. raffinosus* genomes BioSample and GenBank accession numbers are included for reference. Sequence length in base pairs, GC content and number of coding sequences (CDS) are reported per assembly contig. Plasmid replicon type and antimicrobial resistance genes (ARGs) predicted are listed per contig. Pathogen probability was determined as described in the text.

Strain	BioSample accession no.	GenBank accession no.	Type of contig (circular)	Total length (bp)	GC content (%)	CDS no.	Plasmid replicon type	ARGs	Pathogen probability
**Er676**	SAMN30844987	CP104764.1	Chromosome	3 200 986	39.3	3130	repUS43	tetM, ant(9)-Ia, ant(6)-Ia, ermA, efrA, efrB	0.833
		CP104765.1	Plasmid	1 144 253	40	1115	na	IsaA	
**ATCC49464**	SAMN30844988	CP104762.1	Chromosome	3 274 514	39.2	3218	na	efrA, efrB	0.795
		CP104763.1	Plasmid	1 021 776	39.7	992	na	IsaA	
**CX012923**	SAMN20857173	CP081846.1	Chromosome	2 826 834	39.4	2729	na	tetS, ugd, efrA, efrB	0.683
		CP081847.1	Plasmid	984 817	40	957	na	IsaA	
**F162_2**	SAMN18631319	CP072888.1	Chromosome	3 032 044	39.5	2880	repUS43	optrA, fexA, tetM, efrA, efrB	0.825
		CP072889.1	Plasmid	1 186 145	39.9	1115	na	IsaA	
		CP072890.1	Plasmid 2	37 686	33.9	43	repUS1	dfrG, IsaE, IsaB, ermB, InuB, aph(3')-III, ant(6)-Ia	

na, Not applicable.

Additional *Enterococcus* species were selected to assess phylogenetic relationships among the genus. Genomes included: *E. faecalis* V583 (*SAMN02603978*), *E. faecalis* MMH594 (*SAMN00809212*), *E. faecalis* KUB3006 (*SAMD00113788*), *E. faecalis* HA-1 (*SAMN11959349*), *E. faecium* 1 231 501 (*SAMN02595269*), *E. faecium* Dallas 5 (*SAMN17038066*), *E. faecium* ATCC700221 (*SAMN04488250*), *E. gallinarum* FDAARGOS_163 (*SAMN03996308*), *E. gallinarum* EGM181 (*SAMN14408940*), *E. gallinarum* 674 (*SAMN23424745*), *E. casseliflavus* SP11 (*SAMN13955256*), *E. casseliflavus* FDAARGOS_1122 (*SAMN16357291*), *E. casseliflavus* EC-369 (*SAMN10174870*), *E. avium* G-15 (*SAMD00177106*), *E. avium* FDAARGOS_184 (*SAMN04327392*) and *E. avium* 352 (*SAMN10475309*).

### Strain isolation and species identification

*E. raffinosus* strain Er676 was isolated from clean-catch mid-stream urine collected from a consenting woman with a history of recurrent UTI as part of IRB-approved studies 19MR0011 (UT-Dallas) and STU 032016-006 (UT Southwestern Medical Center). Er676 was isolated by plating urine on chromogenic agar (Chromagar Orientation, BD) and picking well-isolated colonies with enterococcal morphology. For species identification, single colonies were selected and subjected to PCR and Sanger sequencing of the conserved 16S rRNA gene as described previously [19, 20]. Sequences were queried against the nr/nt database using megablast (blast v2.10.0) and the species *E. raffinosus* was confirmed [21]. ANI analysis (see sequence alignment methods) of the whole genome sequence further confirmed species assignment.

### Genomic DNA isolation and sequencing

In preparation for sequencing, genomic DNA was isolated from overnight cultures of Er676 and ATCC49464 grown in Brain Heart Infusion (BHI) broth at 37 °C using the DNeasy Blood and Tissue kit (Qiagen). To facilitate lysis, 20 mg ml^−1^ lysozyme and 450 kUml^−1^ mutanolysin were included in the lysis buffer [19, 22]. DNA was quantified via a Qubit fluorometer and quality and integrity were assessed by agarose gel electrophoresis. Next-generation sequencing was performed as previously described using both Illumina NextSeq 500 and Oxford Nanopore Technologies (ONT) MinION following library preparation [19].

### Hybrid genome assembly

Hybrid genome assembly was performed as described previously [19]. Briefly, raw sequence read data were subject to quality assessment and trimming using CLC Genomics Workbench v12.0.3, NanoStats v1.2.0 and NanoFilt v2.6.0 [23]. Illumina reads of quality >20 and length >15 bp and ONT reads of quality >7 and length >200 bp were retained for assembly. Hybrid assembly using reads from both sequencing technologies was performed using Unicycler v0.4.8 [24]. Er676 was assembled at default parameters whereas ATCC49464 was assembled using bold mode. The generated assemblies of both isolates were complete including a circular chromosome and circular megaplasmid in each (Table 1). Where *dnaA* or *repA* was identified, circular sequences were rotated to the starting base of this gene. The completeness and quality of genome assembly generated in this study were assessed using QUAST v5.0.2, Bandage v0.8.1 and BUSCO v1 with the bacterial orthologue set on the gVolante server v1.2.1 [25–27]. Completeness was also assessed using the CheckM v1.0.18 lineage workflow on the Kbase server and CheckM v1.2.2 taxonomy workflow using the *Enterococcus* species detailed in the Genome Collection section of the Methods to construct a marker gene set for the genus [28, 29]. Genome sequences were annotated upon submission using NCBI’s PGAP v4.11 pipeline and prokka v1.14.6 [30, 31].

### Pangenome and phylogenetic analyses

Pangenome estimations and core gene alignment were completed using Roary v3.13.0 with mafft alignment at default parameters [32]. A maximum likelihood phylogeny of 11 *E. raffinosus* isolates was inferred using IQ-TREE v2.2.0.3 with ultrafast bootstrapping (1000 inferences) using the GTR+F+R3 model following model selection with IQ-TREE [33–35]. The phylogenetic tree was minVAR rooted using FastRoot v1.5 and visualized in iTOL [36, 37]. A phylogenetic tree of a subset of diverse *Enterococcus* species was reconstructed similarly, using the GTR+F+I+I+R3 model. Pangenome Venn diagrams were visualized using InteractiVenn [38].

### Functional annotation and enrichment analysis

Prediction of prophage sequences was conducted using PHASTER with default parameters [39]. Further gene predictions were conducted using tools available on the Center for Genomic Epidemiology web server. PlasmidFinder v2.1 was used to identify plasmid replicon sequences using the Gram-positive database at default identity and coverage thresholds [40, 41]. ARGs and known resistance mutations (PointFinder) were identified using ResFinder v4.1 both with the *E. faecalis* and ‘Other’ databases at default identity and coverage thresholds [42–44]. ABRicate v1.0.1 was used with the CARD database at 50 % identity and coverage cutoffs and with the VFDB database at default cutoffs to further query the genomes for antimicrobial resistance and virulence genes [45–47]. PathogenFinder v1.1 with the Firmicute model was used to predict the probability of complete assemblies being a human pathogen [48]. Insertion sequence (IS) elements were identified using ISFinder blastn at standard configuration [49]. Annotation of Clusters of Orthologous Genes (COG) categories was performed on the pangenomes of the chromosome and megaplasmid using EggNOG-mapper v2.0.1 with default parameters [50]. Genes excluded from output did not have any hits. Genes with multiple assigned categories were counted within each group to which they were assigned. COG data counts were normalized to total pangenome size. Specifically, COG gene counts were normalized to the respective total pangenome size, and to test for enrichment, this proportion was compared between the chromosome and megaplasmid using Fisher’s Exact one-tailed post-hoc test with power >0.8 and alpha <0.05.

### Sequence alignments

ANI was assessed using OrthoANI Tool (OAT) v0.93.1 [51]. Genome atlases were generated using blast Ring Image Generator (BRIG) v0.95 with blastn with default parameters only for complete genome assemblies [52]. Overall query coverage and percentage identity were assessed by blastn alignments of each query to the reference. The reference sequence used as template was selected based on size, with the largest sequence being chosen. Genes within variable regions were queried against complete whole genome sequences for identification of alternative alleles in distinct loci using Geneious Prime v2022.1.1 blastn with default parameters. Prophage sequences were compared using EasyFig v2.2.2 with tblastx alignment with standard configuration [53].

## Results

### *E. raffinosus* genome architecture is defined by the presence of both a chromosome and a conserved megaplasmid

**Fig. 1.**
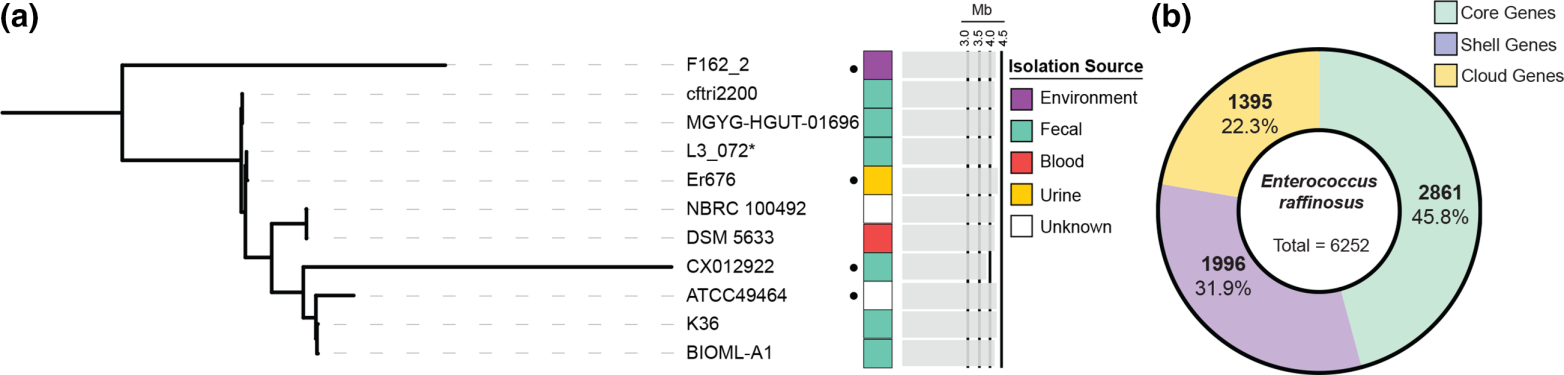
Phylogenetics and pangenome of *Enterococcus raffinosus *. (**a**) Maximum likelihood minVAR-rooted phylogenetic tree of a core gene alignment of 11 *E. raffinosus* genomes. Isolation source of each isolate is depicted by coloured blocks and genome size is denoted by the grey bar graph. Complete genomes are denoted using a black circle. (**b**) Pie chart of pangenome size of *E. raffinosus* inferred from all publicly available genomes. Gene count is annotated along with percentage of the total pangenome. Core genes are found in all isolates, shell genes are found in 15–95 % of isolates, and cloud genes are found in <15 % of isolates. Total pangenome size is listed in the pie centre. *Isolate name was shortened for simplicity.

Reported genome sizes of *E. raffinosus* range from 3.81 to 4.35 Mb [12, 13, 54–56] (Fig. 1a). The average *E. raffinosus* genome is substantially larger than other commonly studied enterococci such as *E. faecalis* and *E. faecium* which have average genome sizes of 2.94±0.15 and 2.85±0.20 Mb, respectively [57, 58]. Publicly available genome assemblies of *E. raffinosus* are scarce. The majority of assemblies are incomplete and fragmented; many are assembled from metagenome sequencing. We therefore sought to increase the number of available *E. raffinosus* genomes by generating a complete genome assembly of a recent urinary isolate, Er676. We observed that the urinary strain Er676 had a total genome size of 4.35 Mb that included a megaplasmid of 1.14 Mb. At the time of this assembly, no complete *E. raffinosus* genome was publicly available, so ATCC49464 was sequenced and used as a reference to validate the genome architecture of *E. raffinosus *. Hybrid assembly of the ATCC49464 genome confirmed that this strain also possessed a megaplasmid (1.02 Mb), supporting the conclusion that the megaplasmid is probably characteristic of this species. Indeed, the recent publication of two *E. raffinosus* complete genomes, F162_2 and CX012922, each containing a similar megaplasmid confirmed this observation [12, 13].

Among the now four available complete genome assemblies, the size of the chromosome of *E. raffinosus* ranges from 2.83 to 3.27 Mb and the megaplasmid size ranges from 0.98 to 1.19 Mb, accounting for 24–28 % of the total genome size. Assessing genome completion using a bacterial orthologue set, both Er676 and ATCC49464 showed 97.4 % coverage of core bacterial genes, missing COG0184 which corresponds to *rpsO*, encoding 30S ribosomal protein S15 [59]. Despite this annotation, we found that *rpsO* is indeed encoded within *E. raffinosus* chromosomes but its sequence identity to the database falls below the detection limit. To further validate this observation, reference assemblies F162_2 and CX012922 were assessed and yielded the same result. At 90 aa long, the *E. raffinosus* 30S ribosomal protein is substantially smaller than the 154 aa long orthologue in the BUSCO database [27]. Additionally, nucleotide megablast queries of the *rpsO* gene from both Er676 and ATCC49464 identified CX012922 and F162_2 as possessing the most similar *rpsO* at 99.3 % identity and 100 % coverage. The overwhelming majority of hits with identity above 85 % belong to diverse enterococcal species (Material S1, available in the online version of this article). Completion assessment of Er676 and ATCC49464 using CheckM indicated a 99.31 completion score using the lineage workflow and a 98.37 completion score using the taxonomy workflow. ANI of Er676, ATCC49464, F162_2 and CX012922 confirms they all belong to the same species with chromosomal identity >97.19 % and megaplasmid identity >96.38 % (Material S2).

To further understand the genetic diversity of the species, we retrieved all *E. raffinosus* assemblies that met our criteria for comparative analysis (genome >3 Mb and ANI >95 %). The core genome phylogeny revealed that clinical strains are divergent from the environmental strain F162_2 (Fig. 1a). Furthermore, the only urinary genome available to date, Er676, is closely related to gut isolates, potentially supporting the hypothesis that urinary enterococci originate from gut populations. Among other *Enterococcus* species, *E. raffinosus* lies in a closely related lineage with *Enterococcus avium* as the most similar relative (Fig. S1). Analysis revealed a pangenome of 6252 genes, of which 2861 (45.8 %) are core genes found in every strain, 1996 were shell genes and 1395 were cloud genes (Fig. 1b). These results suggest that *E. raffinosus* has an open pangenome, and species genetic diversity is present in a relatively large accessory genome.

### Chromosome and megaplasmid genomic diversity among *E. raffinosus* strains

**Fig. 2.**
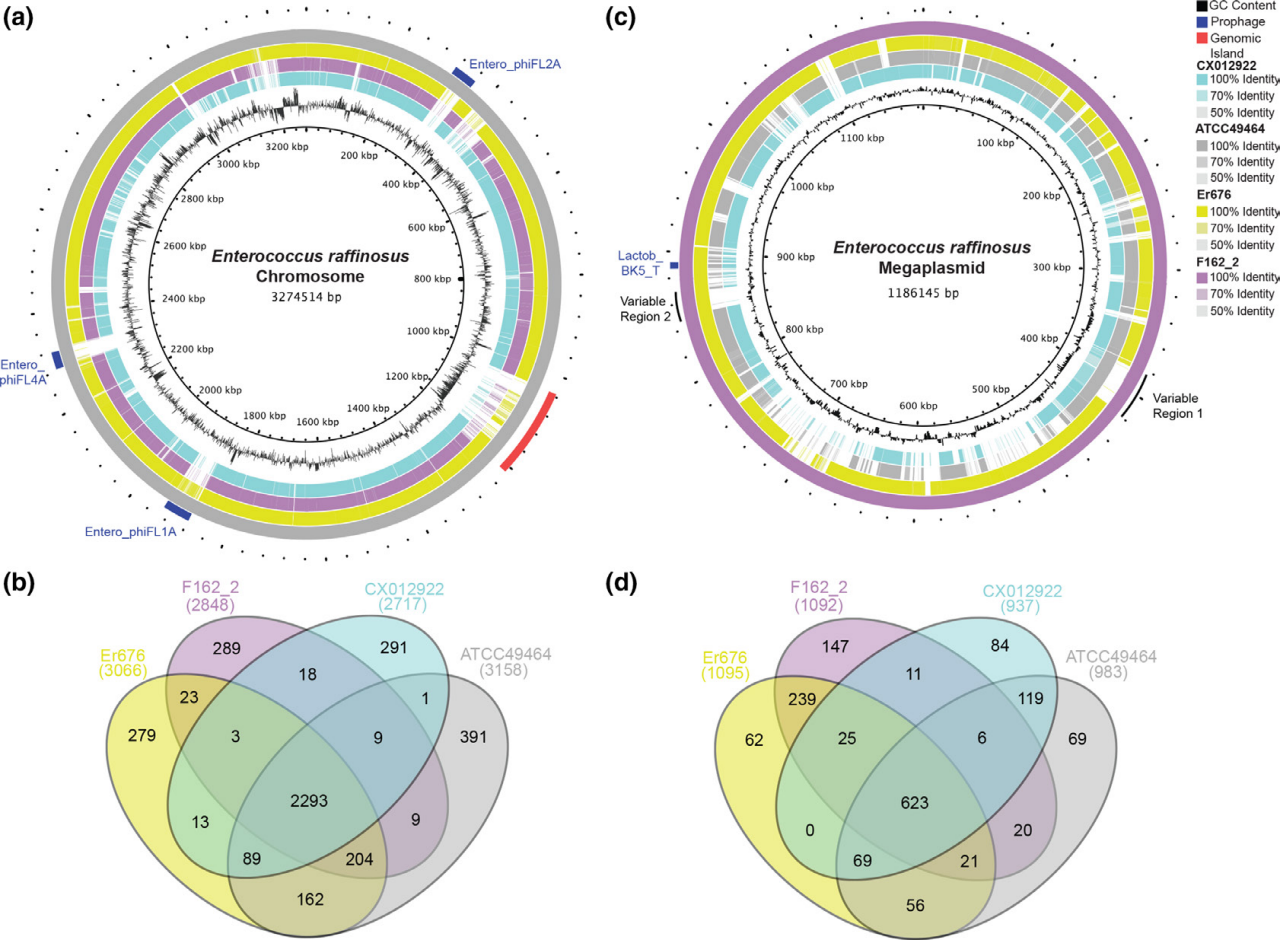
blast atlas of the chromosome and megaplasmid of *E. raffinosus* and their pangenomes. (**a**) blast atlas of the chromosome of complete *E. raffinosus* genomes constructed using ATCC49464 chromosome as a reference. (**b**) Venn diagram of the pangenome of complete *E. raffinosus* chromosomes. (**c**) blast atlas of the megaplasmid of complete *E. raffinosus* genomes constructed using F162_2 megaplasmid as a reference. ATCC49464 (grey), Er676 (green), F162_2 (purple), CX012922 (teal). GC content is shown on the innermost ring in black. Prophage sequences are annotated in dark blue. (**d**) Venn diagram of the pangenome of complete *E. raffinosus* megaplasmids.

To identify large-scale genetic differences between the four *E. raffinosus* genomes, we constructed a blast atlas of the four available complete chromosomes using ATCC49464, possessing the largest chromosome, as a reference. The resulting alignment suggests a high degree of conservation among the strains with regions of low identity corresponding predominantly to intact prophages, their remnants and other transposable elements (Fig. 2a). Our blastn queries of each chromosome and the ATCC49464 reference indicated chromosome coverage >80 % and identity >99.14 %. Specifically, Er676 was 99.77 % identical at 91 % coverage, F162_2 was 99.98 % identical at 85 % coverage, and CX012922 was 99.14 % identical at 80 % coverage. A genomic island region was identified in ATCC49464 that exhibited high genetic variability in the other three strains. The island encodes tRNA-met, a PemK/MazF and an Epsilon-Zeta family Type II toxin–antitoxin system, and transposon Tn552. It is also characterized by a high proportion of IS elements and transposases (Material S3). This island possesses 66.96 and 23.33 % of all IS elements and transposases in the ATCC49464 chromosome, respectively. These findings suggest the island was horizontally acquired at least in part from transposition events and may be a hotspot for genomic evolution (Fig. 2a).

The genomic content of *E. raffinosus* strains was further compared by evaluating the pangenome of the complete chromosomes (Fig. 2b). A total of 4074 chromosomal genes were identified, and a slightly higher percentage of the chromosomal pangenome consisted of core genes (2293, 56.3 %) when compared to the whole-genome pangenome (45.8 %). However, the relatively large accessory chromosomal gene content indicates *E. raffinosus* genetic diversity is not solely determined by the megaplasmid. Each strain possessed between 279 and 391 unique chromosomal genes, with ATCC49464 possessing the largest number of such genes. Additionally, Er676 and ATCC49464 shared 162 genes that were not found in F162_2 or CX012922 (Fig. 2b).

An intriguing genomic feature of *E. raffinosus *, the megaplasmid, contributes to its inflated genome size. Utilizing the largest megaplasmid as reference (F162_2), we created a blast atlas of complete megaplasmids of all four strains. The alignment revealed that many genes were unique to the F162_2 megaplasmid, as indicated by widespread regions of low identity (Fig. 2c). Low-identity regions within the megaplasmid are in part attributed to allelic variations and a mosaic structure of the megaplasmid. Our blastn queries of each megaplasmid to the F162_2 reference showed relatively lower coverage and identity than the chromosome queries. The Er676 megaplasmid was 99.42 % identical at 87 % coverage, the ATCC49464 megaplasmid was 97.31 % identical at 69 % coverage, and the CX012922 megaplasmid was 97.8 % identical at 68 % coverage to the F162_2 megaplasmid. A variable megaplasmid region (variable region 1) located between 378 and 418 kb encoding 29 genes was particularly interesting as it was present in all strains except for Er676 (Fig. 2c). The region codes for a lytic monooxygenase, several hypothetical proteins and tyrosine recombinase xerC, indicating possible phage origins. It further encodes tRNA-ser, glucosyltransferase genes (*glf3, gtfA, gtfB*) and members of the accessory sec (aSec) system (*asp1, asp2, secY_2, secY2, secA_2*). Glucosyltransferases have been implicated in biofilm formation in streptococci [60]. Sec system proteins include SecA2s and accessory Sec proteins (Asps) required for the transport and export of a glycoprotein family that plays a role in adhesion [61]. A blastn search confirmed these genes were also not found within the chromosome of Er676. A secondary region (variable region 2) located between 850 and 930 kb encoding 53 genes was conserved between F162_2 and Er676 megaplasmids but was not conserved in ATCC49464 or CX012922 megaplasmids (Fig. 2c). No clear mechanism for this variability was evident from analysis of genes in the region (i.e. presence of an integrase gene, transposons, etc.). The region contains several hypothetical genes and encodes *pgmB* and *nag3* independently involved in glucose metabolism and biosynthesis [62, 63]. Additional carbohydrate metabolism and transport genes, *malP* and *malH*, specifically involved in maltose transport, are also present in the region [64, 65]. Additionally encoded is *dapE* involved in biosynthesis of lysine, the *phoR* response regulator and *ydaF*, an N-acetyltransferase [66–68]. Lastly, *mta,* a gene involved in transcriptional regulation of various targets, is also present in the region. This gene is reported to regulate, among diverse targets, *bmr* and *blt* which code for multidrug efflux pumps in *Bacillus subtilis* [69, 70]. Of note is that alternative *malH* alleles were found in the chromosomes of ATCC49464 and CX012922.

We then performed pangenome analysis of the complete megaplasmids and identified 1551 genes. Among them, 623 (40 %) were core and 928 were shell; no cloud genes were identified (Fig. 2d). The smaller proportion of core genes relative to the whole genome and chromosome pangenomes indicates that the megaplasmid harbours greater proportional genetic diversity. Among conserved core genes are *agaA* and *rafB* previously reported to encode raffinose metabolism [12, 17]. The largest megaplasmid, found in F162_2 (1.19 Mb), unsurprisingly possesses the largest number of unique genes (147). Interestingly, 239 genes representing unique alleles were exclusive to the F162_2 and Er676 megaplasmids (Fig. 2d). Despite being phylogenetically divergent, Er676 and F162_2 shared the most megaplasmid genes (908 genes) out of any of the four strains.

### The *E. raffinosus* chromosome and megaplasmid harbour diverse prophage

**Fig. 3.**
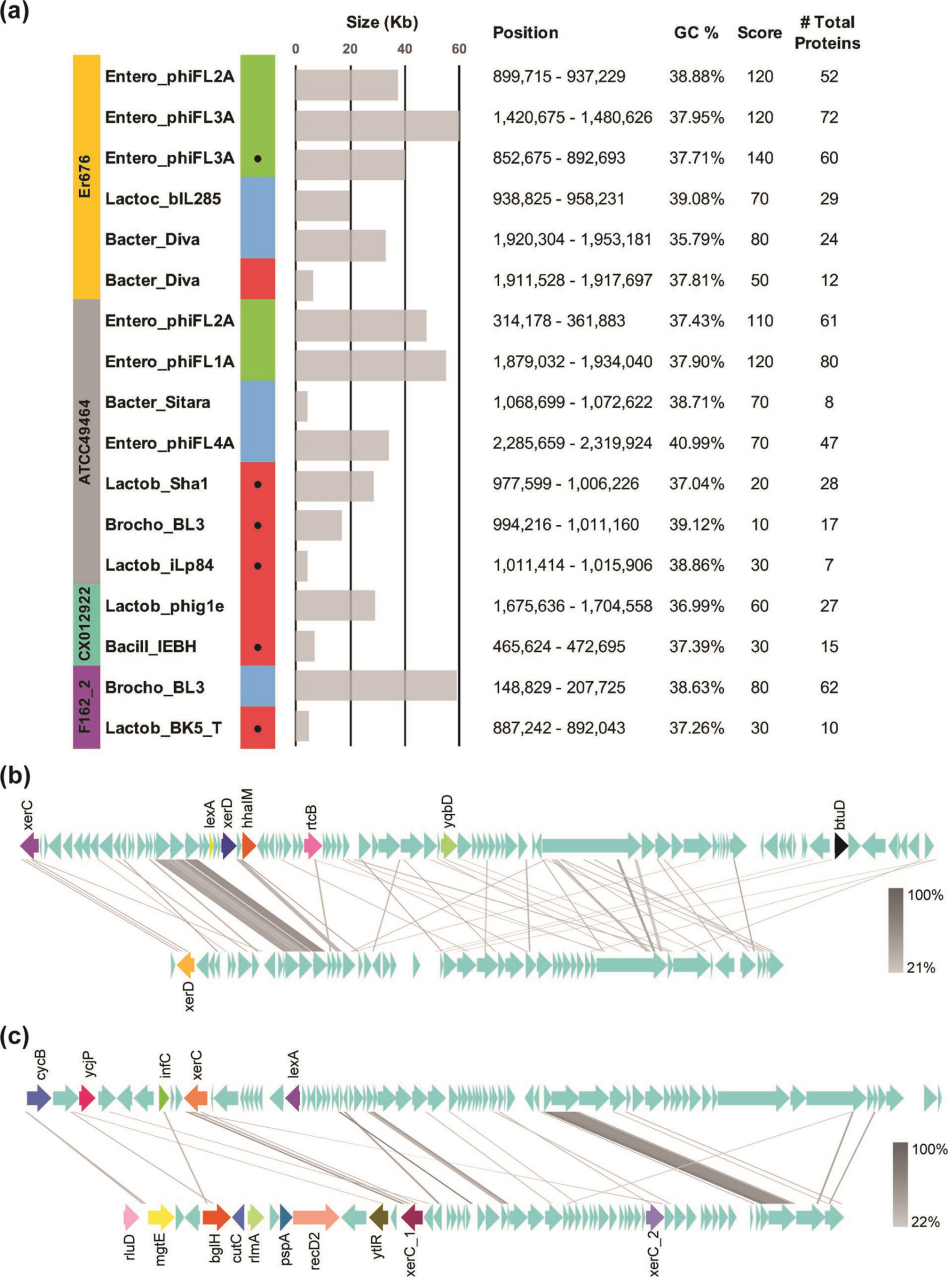
Predictions and tblastx alignment of prophages within complete *E. raffinosus* genomes. (**a**) Prophage sequences predicted to be found within each complete genome are listed. Coloured blocks indicate complete (green), questionable (blue) and incomplete (red) prophages. Circles denote prophages found within the megaplasmid. Bar graph shows total size of the prophage region. Prophage position, GC content, score and number of proteins within the prophage are listed respectively. (**b**) Alignment of intact Entero_phiFL3A prophages identified within the Er676 chromosome and megaplasmid. (**c**) Alignment of intact Entero_phiFL2A prophages identified within Er676 and ATCC49464 chromosomes. Arrows denote coding sequences with known genes arbitrarily coloured and annotated for visualization. Homology lines are shaded based on percentage identity.

Prophages are mobile genetic elements that commonly contribute to bacterial genomic diversity [71]. Utilizing the PHASTER prediction algorithm, we annotated regions of prophage sequences within the four complete *E. raffinosus* genomes to better understand how prophages may contribute to conserved horizontally acquired functions. Intact prophages were identified only in Er676 and ATCC49464 (Fig. 3a). Er676 possesses a total of six prophage sequences, one of which is found within the megaplasmid and is predicted to be intact (Entero_phiFL3A). The remaining chromosomal prophages are Entero_phiFL2A and Entero_phiFL3A which are intact, Lactoc_bIL285, Bacter_Diva which are questionable, and another Bacter_Diva that is incomplete. ATCC49464 possesses a total of seven prophage sequences of which two are intact (Entero_phiFL1A, Entero_phiFL2A) and two are questionable (Bacter_Sitara, Entero_phiFL4A) and localized to the chromosome. The remaining three are incomplete and localized to the megaplasmid (Lactob_Sha1, Brocho_BL3, Lactob_ILp84). CX012922 and F162_2 each possess two prophage remnants, one of which is found within the megaplasmid. The presence of prophage sequences in the megaplasmid is ubiquitous, suggesting the sequence evolution of the megaplasmid is at least partly due to prophage genes. Predicted intact prophages of the same origins were aligned to assess sequence conservation. We aligned the Entero_phiFL2A found in both the Er676 and the ATCC49464 chromosomes, as well as the Entero_phiFL3A from the Er676 chromosome and the Er676 megaplasmid (Fig. 3b, c). In both cases, pairwise sequence identity was low. As suspected from the large variation in prophage sizes, despite having the same annotated name, these prophages were not highly conserved with the exception of a few hypothetical genes. This identifies a limitation of prediction tools in that similarly annotated features do not always have high sequence identity.

### Determinants of pathogenesis and antibiotic resistance are encoded primarily within the *E. raffinosus* chromosome

Despite their association with human infections, enterococci are generally considered commensals of the gastrointestinal tract. Pathogenicity rests in traits that promote virulence, persistence and the ability to overcome immune defences within a host organism [11]. We employed the PathogenFinder prediction algorithm and found that all four complete *E. raffinosus* genomes were predicted to be pathogenic but with variable probabilities [48] (Table 1). The strain most likely to be pathogenic was the urinary isolate Er676 with 0.83 probability and matches to 15 protein families associated with pathogenicity. The strain with lowest pathogenic probability was the gut isolate CX012922 with 0.683 probability and five matches to pathogenic protein families. In all strains, the majority of pathogenic protein matches were found within the chromosome rather than the megaplasmid, suggesting that major pathogenicity determinants may be chromosomally encoded. Queries of the virulence finder database (VFDB) using ABRicate identified a single virulence gene, *fss3,* encoded at two loci in the Er676 chromosome (positions 1981187–1984420 and 3078648–3081819) and one locus in the ATCC49464 chromosome (position 3145396–3148567). All hits were at >98.27 % coverage and >90.92 % identity. This gene encodes a fibrinogen binding protein and, interestingly, fibrinogen accumulation on urinary catheters mediates enterococcal colonization during catheter-associated UTIs [72]. F162_2 and CX012922 did not have any virulence factors as identified by our analysis.

Antimicrobial resistance is another pathogenic trait that increases the complexity of enterococcal infections in clinical settings [3]. Investigation of ARGs and point mutations within the four complete *E. raffinosus* genomes interestingly showed that the megaplasmids encoded a ubiquitous ARG, *IsaA*. Furthermore, all *E. raffinosus* chromosomes possess *efrA* and *efrB*, which encode components of the EfrAB efflux pump. Both Er676 and F162_2 encode multiple other predicted drug resistance genes, specifically four and three ARGs within their chromosomes, respectively. The Er676 chromosome encoded *tetM, ant[9]-Ia, ant[6]-Ia,* and *ermA* resistance genes, which confer resistance to tetracylines, aminoglycosides and macrolides, respectively. The F162_2 chromosome encoded *optrA* (oxazolidinones, phenicol), *fexA* (phenicol) and *tetM* (tetracycline) resistance genes (Table 1). F162_2 additionally encodes a large array of resistance genes on a repUS1 lineage plasmid. Tetracycline resistance genes were found in all except ATCC49464, which does not possess any additional, drug-specific ARGs (Table 1). No known resistance mutations were detected by our analysis in any of the strains.

### The *E. raffinosus* megaplasmid encodes genes involved in transcription and carbohydrate metabolism

**Fig. 4.**
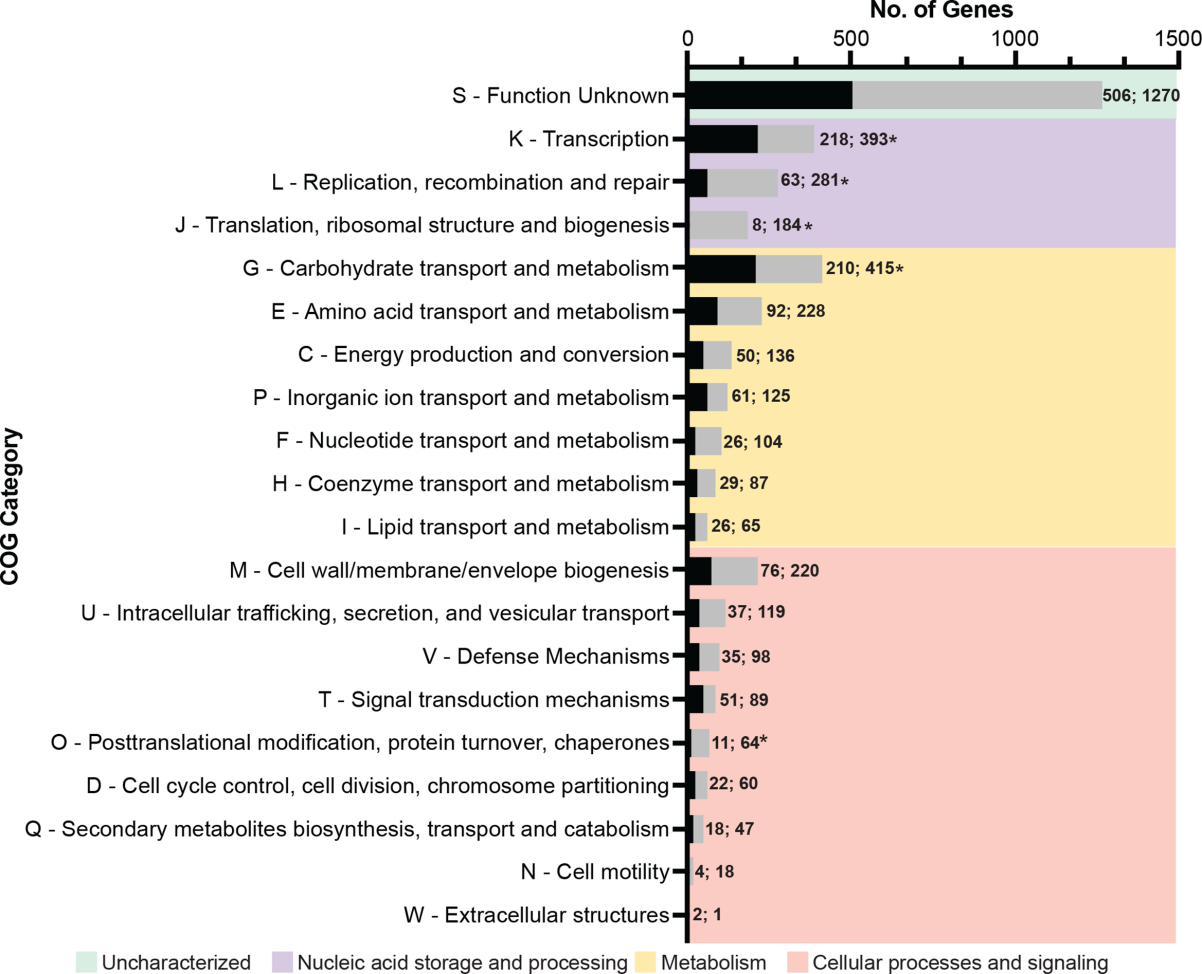
Clusters of Orthologous Genes (COG) functional annotation of chromosome and megaplasmid pangenomes. Functional annotation of all genes in the pangenomes of *E. raffinosus* chromosome and megaplasmids from complete genome assemblies. Bar graph shows all present COG categories in the chromosome (grey bars) and megaplasmid (black bars) superimposed. The total number of genes is annotated respectively at bar ends. Categories were grouped and shared by functional class. *P<0.05 by Fisher’s Exact one-tailed post-hoc test.

Pangenome estimations and shared gene content provide important insight into strain-level genetics. However, an understanding of the functions encoded by these genes is crucial. To further dissect the potential functional role of the conserved *E. raffinosus* megaplasmid, we functionally annotated the complete chromosomal and megaplasmid pangenomes using EggNOGmapper. Annotations revealed that the majority of functional categories were distributed similarly between the chromosome and megaplasmid. Interestingly, we found that COG categories J (Translation, ribosomal structure and biogenesis), O (Posttranslational modification, protein turnover, chaperones) and L (Replication, recombination and repair) may be proportionally enriched in the chromosome (Fig. 4, Material S4). This suggests that the functions of protein translation and DNA replication may be more reliant on chromosomal genes. Of note, COG category J genes relating to translation, ribosomal structure and biogenesis comprised only 0.516 % of the megaplasmid pangenome (eight genes) compared to 4.516 % of the chromosome pangenome (184 genes, *P*=0.0301) (Fig. 4). Previous reports indicated that the megaplasmid of CX012922 lacked an identifiable encoded replication initiation protein, which we also observed in Er676 and ATCC49464 (Table 1) [12]. Together, the observed enrichment of DNA replication genes within the chromosome and the lack of an encoded replication initiation protein in the megaplasmid suggest that he megaplasmid may rely on chromosomally encoded genes for its replication.

Interestingly, we observed two COG categories, G (Carbohydrate transport and metabolism) and K (Transcription), that were potentially enriched in the megaplasmid pangenome. Proportional enrichment of category K, consisting of 14.055 % (218 genes, *P*=0.0461) of the megaplasmid pangenome compared to 9.647 %, suggests the megaplasmid may play an indispensable role in cellular viability and may support adaptation to environmental changes via transcriptional regulatory mechanisms. The possible enrichment of carbohydrate transport and metabolism genes in the megaplasmid was notable with genes in COG category G comprising 13.540 % of the megaplasmid pangenome (210 genes) versus 10.187 % of the chromosome pangenome (415 genes, *P*=0.0462). The megaplasmid encoded carbohydrate utilization genes necessary for the metabolism and transport of raffinose, lactose, fructose, mannose, sorbitol, galactose, maltose and cellobiose.

## Discussion

This study reports the complete assembly of the first urinary *E. raffinosus* strain and complete assembly of clinical type strain, ATCC49464. Particular emphasis was placed on Er676 and comparative analyses of all complete *E. raffinosus* strains available to date. Phylogeny and pangenome analyses of all available *E. raffinosus* genomes, including draft assemblies, reveal a conserved core genome with relatively large accessory genome probably responsible for species diversity. Indeed, large inter-species diversity has been reported for other enterococci and *E. raffinosus* [12, 73]. Much of the chromosomal diversity is due to prophage integration and transposable elements, indicating unique genes are attributed to horizontal gene transfer. Of note is a genomic island region in ATCC49464 encoding a multitude of hypothetical proteins, two Type II toxin–antitoxin systems, and phosphotransferase system (PTS) components. As evidenced by the presence of a transposon, tRNA and a high proportion of insertion sequences, this region probably originated from evolutionary transposition events and is a hotspot for genomic diversity. Additional studies are necessary to further characterize regions of genomic evolution in order to understand both evolutionary pressures and adaptation of *E. raffinosus* to its habitat. This is particularly important in the context of human health. Er676 and ATCC49464 share a collection of unique accessory chromosomal genes not found in other complete genomes. Further characterization of these genes and their potential role in clinical settings is an important future endeavour.

Perhaps the most intriguing genomic characteristic of *E. raffinosus* is the megaplasmid, which appears to be a ubiquitous feature of this species. Both Er676 and ATCC49464 assemblies confirm the presence of the megaplasmid, suggesting it has an important role in the physiology of *E. raffinosus *. The megaplasmid harbours a large accessory genome as well, probably also contributing to the inter-species diversity and adaptability of *E. raffinosus *. Megaplasmid diversity is at least in part due to phage invasion, as evidenced by the detection of incomplete prophages within complete megaplasmid sequences. This suggests the megaplasmid is mosaic. Furthermore, a region of sequence variability among complete megaplasmids with an upper boundary at 358 kb that encodes biofilm formation and adhesion factors is hypothesized to have been acquired from phage integration. Intriguingly, this region is present in all megaplasmids except for the Er676 megaplasmid. While these factors are absent from the urinary megaplasmid, this does not indicate the absence of alternative biofilm- and adhesion-promoting factors. It is also possible these megaplasmid factors are dispensable in the urinary niche. Additionally, a second variable region with an upper boundary at 850 kb was found to be conserved between F162_2 and Er676 but not the other two megaplasmids. This region is not clearly explained by a specific acquisition mechanism but encodes imperative genes that play a role in glucose and maltose metabolism and biosynthesis, response to phosphate limiting conditions, and transcriptional regulation of diverse targets. Their conservation between an environmental and a urinary isolate may suggest their important role in survival and adaptation to distinct and changing habitat stressors such as limited availability of carbohydrate sources.

Although previously characterized as a plasmid, the megaplasmid remains an intriguing genomic composition. Among its genes are those coding for raffinose metabolism, a distinguishing characteristic of the species, as well as other carbohydrate transport and metabolism genes, and transcription genes. This is particularly important in survival and adaptation to different environmental conditions and suggests that the megaplasmid may be key to the growth and colonization of *E. raffinosus *. It can be hypothesized that it has evolved from unique environmental pressures the species encounters in the diverse habitats in which it is found. The megaplasmid does not have a previously characterized replication initiation protein, remains conserved among all complete *E. raffinosus* assemblies available to date, and encodes many vital genes, suggesting it may predominantly function as a second chromosome [74].

We noted that ARGs, with the exception of a single efflux pump encoded by *IsaA*, have not been commonly detected within the complete megaplasmid sequences using the ResFinder and ABRicate pipelines. Additionally, many of the pathogenic protein families predicted to exist in *E. raffinosus* and increasing its probability of pathogenesis in humans, are also not identified within the megaplasmid. These findings further suggest that the megaplasmid potentially plays a role in growth and cellular physiology but not in antibiotic resistance or virulence. Studies of gene essentiality in the *E. raffinosus* megaplasmid can shed further light on the important role of the megaplasmid in its species persistence.

## Supplementary Data

Supplementary material 1

Supplementary material 2
