# Supplementary material for: Inter-species diversity and functional genomic analyses of closed genome assemblies of clinically isolated, megaplasmid-containing Enterococcus raffinosus Er676 and ATCC49464
Source: Access Microbiol. 2023 Jun 12;5(6):acmi000508.v3. doi: 10.1099/acmi.0.000508.v3 (PMC10323788; doi:10.1099/acmi.0.000508.v3)
Supplement: Supplementary material 1 [file acmi-5-508.v3-s001.pdf]

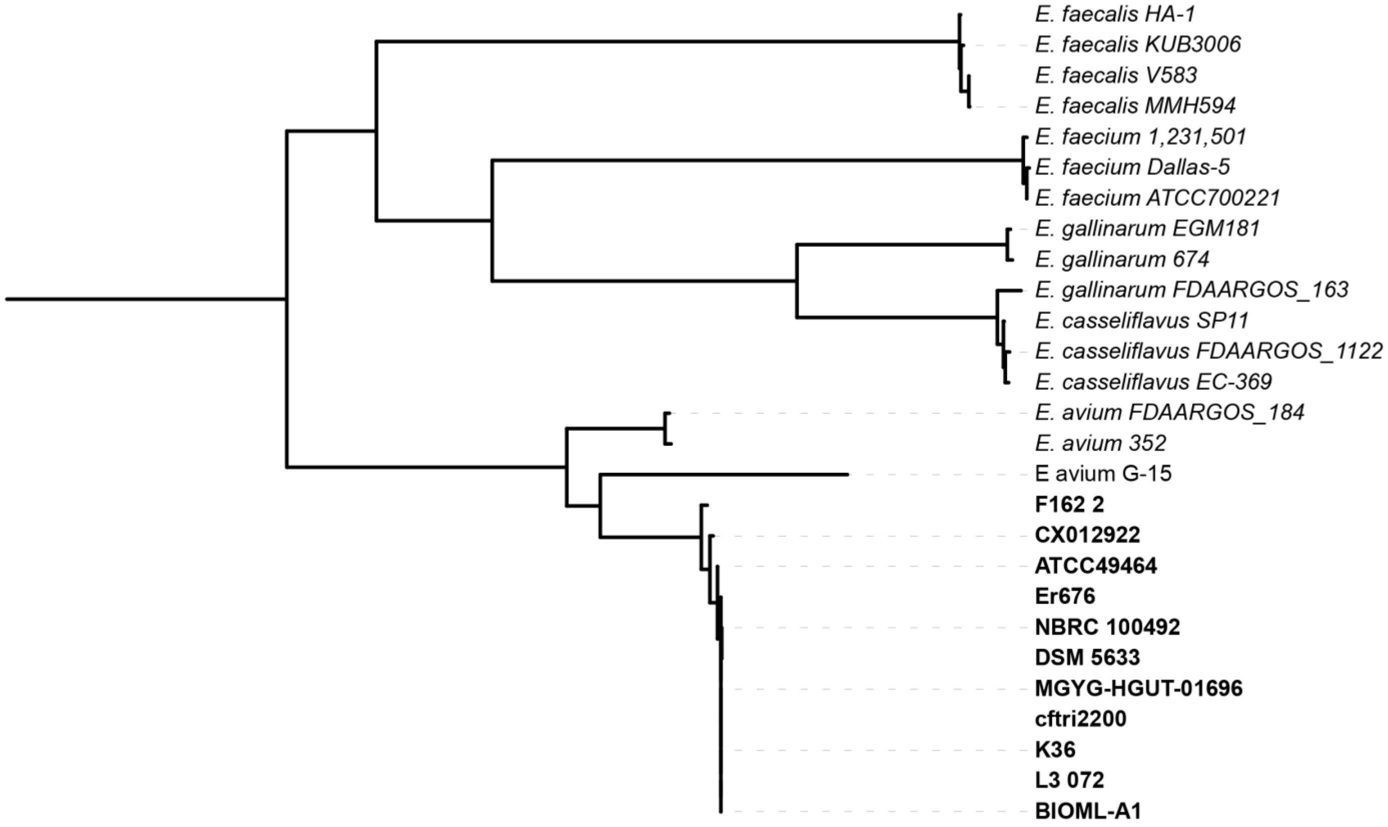

Supplemental Figure 1. Phylogenetics of *Enterococcus raffinosus* among other *Enterococcus* species. Maximum likelihood minVAR-rooted phylogenetic tree of core gene alignment of 27 *Enterococcus spp.* genomes. Species and strain name are listed at leaf ends. *E. raffinosus* strains are indicated in bold.
